# Responsiveness of the single item measure to detect change in physical activity

**DOI:** 10.1371/journal.pone.0234420

**Published:** 2020-06-25

**Authors:** Paul O’Halloran, Michael Kingsley, Matthew Nicholson, Kiera Staley, Erica Randle, Annemarie Wright, Adrian Bauman

**Affiliations:** 1 School of Psychology and Public Health, La Trobe University Bundoora Australia, Bundoora, Australia; 2 Centre for Sport and Social Impact, La Trobe University Bundoora Australia, Bundoora, Australia; 3 Holsworth Research Initiative, La Trobe University Bendigo Australia, Bendigo, Australia; 4 Department of Exercise Science, University of Auckland, Auckland, New Zealand; 5 Victorian Health Promotion Foundation (VicHealth), Carlton, Australia; 6 The University of Melbourne (Honorary), Parkville, Australia; 7 School of Public Health Sydney University Australia, Sydney, Australia; Teesside University/Qatar Metabolic Institute, UNITED KINGDOM

## Abstract

**Objective:**

The objective of this study was to evaluate the responsiveness of the single item measure (SIM) to detect change in PA when compared to hip-worn accelerometry. A secondary aim was to provide further data on validity of the measure at a single time point.

**Methods:**

Validity of the SIM to determine the number of days of ≥30 minutes of accelerometer-derived moderate-to-vigorous physical activity (MVPA) was assessed in 120 participants (78% female, 19% male, 3% other; age: 46 ± 11 years). Analysis of change was based on change in the number of days that participants completed ≥30 minutes of MVPA determined by the SIM and accelerometry over two 7-day periods in 90 participants (age: 47±11 years). Accelerometer data were analysed as total minutes of MVPA per day (MVPA-total) and as sustained bouts of 10 minutes or more of MVPA (MVPA-bouts). Validity of the SIM to detect change in MVPA, using accelerometer data as the reference measurement, was examined through Spearman’s correlation and agreement in classification of change between SIM and accelerometry. Responsiveness to change was assessed by standardised response means and Cohen’s d.

**Results:**

Standardised response means for PA change were moderate for the SIM (0.77), MVPA-total (0.57) and MVPA-bouts (0.79). The correlation for change in number of days ≥30 minutes MVPA between the SIM and accelerometry were small to moderate (MVPA-total: r = 0.36 and MVP-bouts: r = 0.40). The SIM displayed moderate accuracy (60%-63%), using accelerometer data as the reference measurement, in detecting increases in days ≥30 minutes of MVPA.

**Conclusion:**

The SIM is a potentially useful assessment tool for evaluating change in MVPA, particularly when device-based measures or longer self-report measures are not feasible.

## Background

Measurement of physical activity (PA) is important at a population level and for examining the effectiveness of large-scale interventions and programs designed to increase physical activity participation [1, 2]. Device-based measurement of PA using accelerometers is often the chosen method given supportive evidence of the validity and reliability of these instruments for assessing PA [3, 4]. However, the use of accelerometers is not always feasible due to expense, practical constraints, and poor compliance associated with these devices [5–7]. Consequently, self-report measures remain an option for measuring PA in large populations and for evaluation research [8–10]. Meta-analyses regarding the validity of self-report measures suggest that there is no clear best self-report measure of PA [11], validity of most measures is modest [12] and further work is required to determine the responsiveness of measures for assessing change in PA [3, 11]. In addition, the intended use of the measure and the feasibility of administration should be considered when selecting a self-report measure [3, 19].

Single item measures of PA have been developed to assess the physical activity of population segments, including older adults [13, 14, 15] and adolescents [16] in settings where time and resources are limited. The single item measure (SIM) developed by Milton and colleagues [1] has been used widely to assess the PA of adults [17, 19] and asks respondents to report the number of days in the last week that they undertook at least 30 minutes of moderate to vigorous physical activity (MVPA) related to leisure or transport. Given the SIM can provide a parsimonious measure of activity, it has been used to assess population levels of PA [17, 18] and the effectiveness of programs designed to increase PA [19]. There is emerging supportive evidence for the measurement properties of the SIM, that includes test-retest reliability [1] and concurrent validity, based on comparisons with accelerometer data [2, 20]. Although Milton et al. [20] reported a slightly larger correlation between the SIM when accelerometer data were analysed in 10-minute bouts of MVPA per day (r = 0. 57 MVPA bouts vs. r = 0.46 MVPA total), Wanner et al. [2] reported a considerably larger correlation when the SIM was correlated with total minutes of MVPA per day (r = 0.15 MVPA bouts *vs*. r = 0.44 MVPA total). Consequently, one issue that has yet to be resolved is whether correlations between the SIM and accelerometer data are higher when accelerometer data are analysed in 10-minute bouts of MVPA per day or total minutes of MVPA per day. Thus, further research of the SIM is warranted.

If PA self-report measures are to be used in evaluation research or for PA surveillance, it is essential that they provide valid assessments of change [3, 19, 21, 22]. In the last 10 years studies have begun to assess the validity of self-report measures to detect change in MVPA using accelerometer data as the reference measurement [19, 23–29]. In one of the earliest of these studies, moderate agreement (r = 0.52) was reported between the 16-item Global Physical Activity Questionnaire (GPAQ) [30] and accelerometer data when the extent of change in physical activity was compared over two 7-day periods [23]. Although these authors reported the GPAQ to be a valid measure of change in MVPA, the conclusion was based on just 21 participants with valid accelerometer data over a minimum of 5 days [23]. More recently, the short 6 x 2-part REGICOR [27] questionnaire was reported to correlate significantly with changes in moderate intensity activity over a 4-month period as detected by accelerometer data when analysed in 3-minute (r = 0.34) and 10-minute bouts (r = 0.26). Although these data are promising, the REGICOR may not be well-suited to assess change in all settings as it requires participants to respond to 12 items using a one-month reference period. A further study attempted to measure the validity of two brief self-report PA measures to measure change against accelerometer data; however, no conclusions could be drawn given that 91% of the participants experienced no change over the two assessment periods [19].

A more recent trend has been to examine the responsiveness of self-report measures to detect change in MVPA using accelerometer data as the reference measurement under conditions where change in MVPA is being actively promoted [24–26, 28, 29]. Several studies reported that the IPAQ-SF [31] and accelerometer were both able to detect change in physical activity as a result of interventions designed specifically to increase activity [24, 26, 28]. However, these findings are difficult to interpret given changes in physical activity detected by the IPAQ were not significantly correlated with change detected by accelerometer in two studies [24, 28]. Other studies are difficult to generalise given that they related specifically to cardiac patients [24] or did not include any men in their samples [24, 28]. Limb et al. [26] examined the ability of the IPAQ-SF and the General Practice Physical Activity Questionnaire (GPPAQ) [32] to assess change relative to accelerometry in a large community-based intervention designed to increase walking in a relatively diverse sample of men and women. Findings from this study suggest limited utility of these measures to detect change with only the walking questions of the IPAQ providing a reasonable, albeit less precise, estimate of the changes detected by accelerometer.

Stronger evidence of responsiveness has emerged from Vandelanotte et al. [29] who examined the responsiveness of the 8-item Active Australia Survey [33] to detect change in PA against accelerometer data and reported a moderate correlation (r = 0.36) between residual scores on the measure and accelerometer data for change in MVPA over a 3-month period. Although the investigators raised concerns about the overall validity of the measure, they concluded that the Active Australia Survey was responsive to change in some population subgroups [29]. It has been noted that correlational analyses are not the preferred method for assessing responsiveness of PA measures for detecting change [25]. Instead, some recent investigations have included effect size statistics, such as standardised response mean and Cohen’s d, for assessing responsiveness of PA measures to change [25, 34]. The responsiveness of Active Australia Survey (AAS), the United States National Health Interview Survey (USNHIS) walking for exercise items, and accelerometry (Actigraph GT1M) have been evaluated using effect size statistics in an intervention designed to increase PA in people living with diabetes [25]. All three measures of PA were found to have modest responsiveness to change and it was concluded the two self-report measures were relatively comparable in responsiveness to the device-based measure [25]. Given the lack of consistency in findings and relative paucity of studies that report responsiveness, more research pertaining to the responsiveness of self-report measures to measure change in MVPA is required. Also, no study has examined the responsiveness of the widely used SIM to detect change in MVPA using accelerometer data as a reference measurement. Notwithstanding the limitations of accelerometry that preclude this as being used as a gold standard assessment of MVPA [5,27,35], accelerometers are widely used in population PA surveillance and are typically rated as preferable to self-report measures for most purposes [3, 36, 37].

Therefore, the primary aim of the study was to assess the responsiveness of the SIM to detect change in MVPA, using accelerometer data as a reference measurement, in a study where change in MVPA was being actively promoted. A secondary aim was to provide further data on the validity of the SIM compared to accelerometer data for assessing the number of days ≥30 minutes MVPA at a single time point.

## Methods

### Participants

Staff, and their cohabitants, from a regional campus of a University in Australia were invited to participate via an advertisement in an electronic University newsletter. To be eligible, participants were required to be over 18 years of age and needed to have passed screening with the Physical Activity Readiness scale [38]. In total, 128 participants provided informed consent and the study was approved by La Trobe University Human Ethics Committee (ref: E15-121).

### Procedure

All participants were invited to attend four face-to-face sessions to instruct them in the program, fit the accelerometer and complete the questionnaire. At the first, which served as the information session, participants completed a demographic questionnaire and were provided with a triaxial accelerometer (GT3X+; Actigraph LLC, USA) that was fitted to a belt so that the device was placed superior to the person’s right hip. Accelerometers were calibrated according to the manufacturer’s guidelines and set to record triaxial accelerations at 100 Hz [39]. Participants were instructed to wear the device during waking hours, except when in water, for two 7-day data collection periods.

Week one: In the first of these data collection periods, participants were instructed to maintain their usual routines wherever possible. At the completion of this first 7-day period participants returned the accelerometer at the second face-to-face session and completed the SIM, which asks participants: *In the past week*, *on how many days have you done a total of 30 minutes or more of physical activity*, *which was enough to raise your breathing rate*? *This may include sport*, *exercise*, *and brisk walking or cycling for recreation or to get to and from places but should not include housework or physical activity that may be part of your job*. Participants then had one week with no monitoring before returning for the third face-to-face session in which they were fitted with the accelerometer again for the second 7-day assessment of activity.

Week two: Several intervention components were delivered to encourage change in physical activity during the second 7-day period of physical activity monitoring: participants were asked to increase their walking and/or running during this period; were invited to participate in a “step towards summer physical activity challenge”; and were offered access to regular supervised group walking sessions that were supervised by a personal trainer. The activity challenge offered prizes to the individuals and teams (if that was the preferred option) with the largest increase in accelerometry-derived step counts between assessments. At completion of this 7-day period participants retuned for the fourth and final time to return the device and complete the SIM for a second time.

### Data analysis

Acceleration recordings were analysed using the manufacturer’s software (Actilife version 7.0; Actilife Corp., USA). The vector magnitude, in 1-minute epochs, was used to determine non-wear time according to the Choi wear time validation algorithm [1, 40]. Valid wear time data were classified in 1-minute epochs as being MVPA using the Freedson Adult VM3 algorithm [41] where vector magnitude >2690 counts per minute were classified as MVPA. Accelerometry wear time minimums for inclusion for data analysis were 8 hours per day for a full 7-days. The rationale for the minimum of 7 valid days of wear time was that the SIM assesses MVPA over a 7-day period. In line with the convention adopted in other investigations [2, 20] the accelerometry criterion of the number of days of meeting ≥30 minutes of activity was calculated in 2 ways. The total time spent in MVPA each day was calculated regardless of bout duration (MVPA-total) and also with respect to MVPA per day in sustained bouts of 10 minutes or more (MVPA-bouts). The primary rationale for inclusion of bouted activity as a method for analysing accelerometer data relates the potential utility of the SIM for examining PA change in community settings where a focus is often on increasing physical activity in bouts of 10 or more minutes (such as the active 10 program in the UK [42]and participation in other organised sport-based activities).

### Statistical analyses

**Fig 1:**
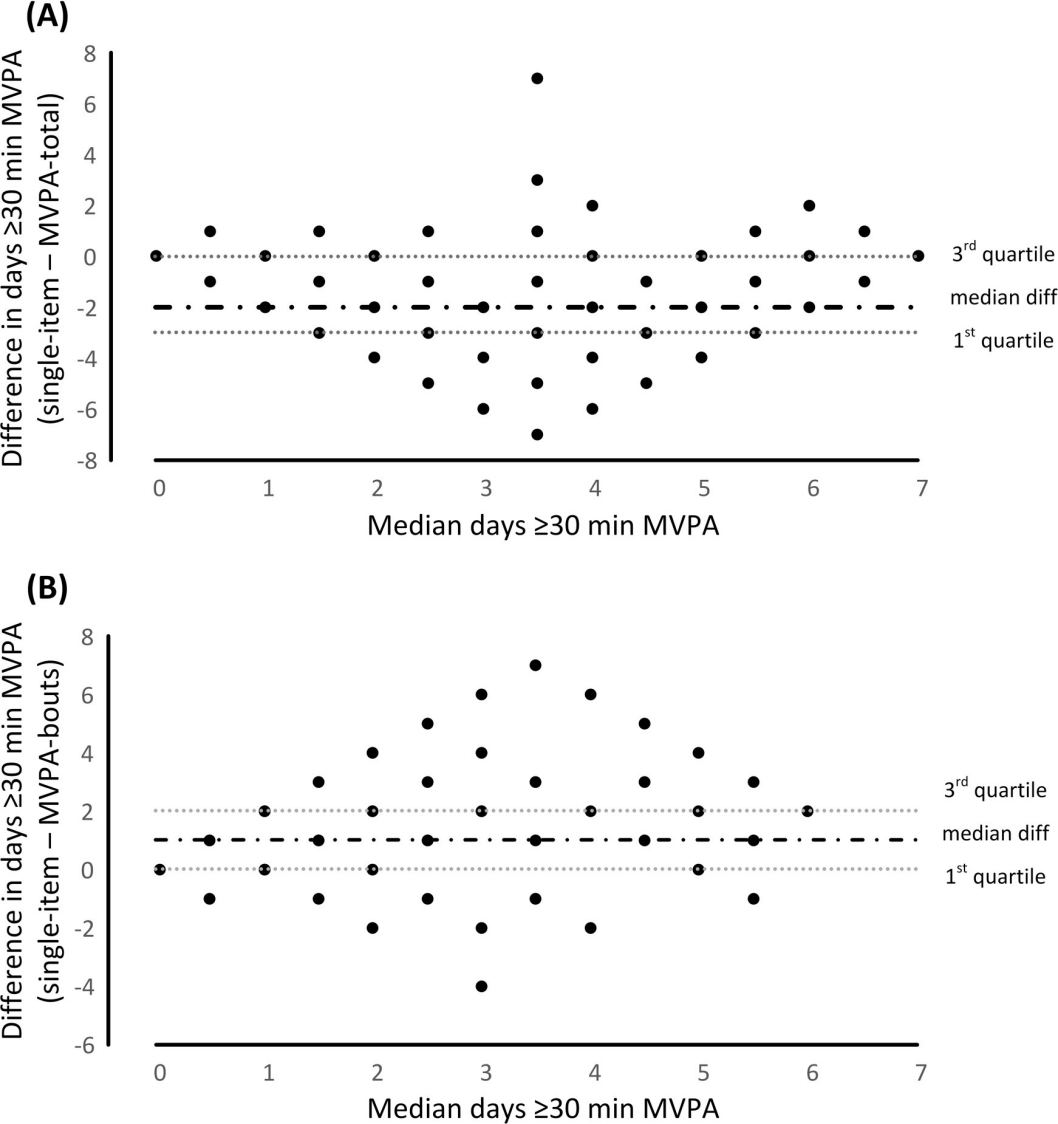
Difference in days ≥30 minutes of moderate-to-vigorous intensity physical activity (MVPA) between the single item measure (SIM) and accelerometry: (A) total MVPA (MVPA-total) and (B) MVPA occurring in bouts (MVPA-bouts). Median difference and inter-quartile range for the ordinal distributions are presented as dotted horizontal lines against the median of the two measures (single-item and accelerometer).

Statistical analyses were performed using IBM SPSS Statistics for Windows (Version 25; IBM Corporation, USA). Given that the unit of measurement in this study was days of ≥30 minutes MVPA and data were not normally distributed, group data are presented as medians and interquartile range (IQR). Spearman’s rank correlations were calculated in order to assess relationships between SIM and accelerometer data (MVPA-total and MVPA-bouts). K statistics and percent agreement were used to determine the level of relative agreement between the SIM and accelerometer data. Absolute agreement between SIM and accelerometer data (MVPA-total and MVPA-bouts) in determining days of ≥30 minutes MVPA was evaluated by plotting the difference in days (SIM minus accelerometer) against median days of ≥30 minutes MVPA from SIM and accelerometer. As the distribution of the residuals did not display significant heteroscedasticity, median and IQR for these ordinal distributions were calculated and presented in Fig 1.

The responsiveness and validity of the SIM to detect change in MVPA were examined using accelerometer data as the reference measurement. Overall estimates of response to change (effect sizes) for the SIM and accelerometer data were obtained using the standardised response mean (SRM) and Cohen’s d. SRM was calculated by dividing the mean change in the number of days ≥30 minutes MVPA (i.e., follow-up minus baseline) by the standard deviation of individual change scores. Cohen’s d was calculated as the mean of individual change scores divided by the pooled standard deviation of the scores during baseline and follow-up. These effect size calculations can be interpreted in line with the following criteria: less than 0.20 trivial, 0.20 to less than 0.50 small, 0.50 to less than 0.80 moderate and greater than or equal to 0.80 large [43]. The validity of the SIM to detect change, using accelerometry as the reference measurement, were also assessed through several additional measurements of agreement. Association and inter-measurement reliability between the change in the number of days ≥30 minutes MVPA (week 2 –week 1) as recorded by SIM and accelerometer data were calculated using Spearman’s rank correlations and k statistic, respectively. In addition, we determined agreement between SIM and accelerometry (MVPA-total and MVPA-bout) to classify change in PA as either “increased”, or “decreased/no change”. The decision to combine those who decreased with those who did not change into a single category (decreased/no change) was based on the study design, where participants were encouraged to increase their PA during week 2 and a small number of participants decreased their PA.

## Results

Of the 128 people recruited to this study, data from 8 participants (6%) did not meet the criteria for analysis. In total, 120 participants (94% valid responses at baseline; 94 females, 23 males, 3 others) were included in analyses at a single time point (age: 46 ± 11 years, stature: 168 ± 8 cm, body mass: 76.2 ± 14.2 kg, body mass index: 27.1 ± 4.4 kg/m^2^). Valid data were available for both weeks for 90 participants (70% valid responses at baseline and follow-up; 71 females, 18 males, 1 other; age: 47 ± 11 years, stature: 167 ± 8 cm, body mass: 74.2 ± 14.5 kg, body mass index: 26.7 ± 4.7 kg/m^2^) and these data were included in analyses of change in physical activity from week one to week two.

### Agreement between SIM and accelerometry at a single time point

Table 1 provides frequency data of days with ≥30 minutes of MVPA. Bivariate correlations for the number of days ≥30 minutes MVPA between the SIM and accelerometry were r = 0.22 (p = 0.037) and r = 0.51 (p < 0.001) for MVPA-total and when MVPA-bouts, respectively. The median difference between the number of days with ≥30 minutes of MVPA between the SIM and accelerometry was -2 days (IQR: -3 to 0 days) for MVPA-total (Fig 1A) and 1 day (IQR: 0 to 2 days) for MVPA-bouts (Fig 1B).

**Table 1:** Frequency of days with ≥30 minutes of moderate-to-vigorous intensity physical activity (MVPA) at a single time point as measured using accelerometry and single item measure (SIM) self-report.

Days of ≥30 min MVPA	Accelerometer MVPA-total n (%)	Accelerometer MVPA-bouts n (%)	Self-report SIM n (%)
0	4 (3.3%)	47 (39.2%)	18 (15.0%)
1	4 (3.3%)	27 (22.5%)	21 (17.5%)
2	14 (11.7%)	22 (18.3%)	19 (15.8%)
3	13 (10.8%)	8 (6.7%)	23 (19.2%)
4	18 (15.0%)	8 (6.7%)	13 (10.8%)
5	20 (16.7%)	7 (5.8%)	12 (10.0%)
6	27 (22.5%)	1 (0.8%)	5 (4.2%)
7	20 (16.7%)	0 (0%)	9 (7.5%)
Median	5.0	1.0	3.0
IQR	3.0–6.0	0–2.0	1.0–4.0

The number of people achieving five days or more ≥30 minutes MVPA via accelerometer for MVPA-total and MVPA-bouts as well as via the self-report were 67 (56%), 8 (7%) and 26 (22%), respectively.

### Ability of the SIM to detect change in physical activity

The median change in number of days ≥30 minutes MVPA from week one to week two via accelerometer for MVPA-total and MVPA-bouts as well as via the self-report were 1 day (IQR: 0 to 2), 1 day (IQR: 0 to 3) and 2 days (IQR: 0 to 3), respectively. The majority of participants (MVPA-total: n = 53 (59%), MVPA-bouts: n = 58 (64%)) increased the number of days that they achieved ≥30 minutes MVPA (Table 2).

**Table 2:** Binary classification tests for the single item measure versus accelerometer total MVPA (MVPA-total) and MVPA occurring in bouts (MVPA-bouts) for detecting increases in number of days ≥30 minutes MVPA from week one to week two.

	Detecting increases in number of days ≥30 min MVPA	
	Single item *vs* accelerometer (MVPA-total)	Single item *vs* accelerometer (MVPA-bouts)
Participants in category (%)	59	64
Accuracy (%)	60.0 (49.1 to 70.2)	63.3 (52.5 to 73.3)
Sensitivity (%)	63.1 (50.2 to 74.7)	69.2 (56.6 to 80.1)
Specificity (%)	52.0 (31.3 to 72.2)	48.0 (21.1 to 68.7)
Positive predictive value (%)	77.4 (68.6 to 84.3)	77.6 (69.7 to 83.9)
Negative predictive value (%)	35.1 (24.9 to 47.0)	37.5 (25.8 to 50.9)

Responsiveness was moderate to high for the SIM and accelerometry MVPA-total and MVPA-bouts (all ≥ 0.57). Notwithstanding overlapping confidence intervals across all three measurements, the SRM value for the SIM (0.77; 95% CI: 0.45–1.01) was more similar to MVPA-bouts (0.79; 95% CI: 0.58–0.99) than the MVPA-total (0.57; 95% CI: 0.33–0.77). Consistent with this, there was greater similarity between the Cohen’s d values for change between the SIM (0.82; 95% CI: 0.54–1.05) and MVPA-bouts (0.81; 95% CI: 0.61–1.06) than between the SIM and MVPA-total (0.61; 95% CI: 0.36–0.82).

The correlation for change in number of days ≥30 minutes MVPA between the SIM and accelerometery MVPA-total was r = 0.36 (p < 0.001), while the correlation between the SIM and accelerometer MVPA-bouts (MVPA-bouts) was r = 0.40 (p < 0.001). The overall accuracy of the SIM, relative to accelerometry as the reference measurement, in assessing increases in number of days ≥30 minutes MVPA from week one to week two was similar for MVPA-total and MVPA-bouts. In terms of identifying participants who had increased PA (sensitivity), the SIM correctly detected over 60% of people who increased their MVPA. The percentage of people who self-reported increasing PA with the SIM who actually did according to accelerometry (PPV) was 78% and 77% for MVPA-total and MVPA-bouts, respectively. In terms of specificity, the SIM correctly detected approximately 50% of people who did not increase (i.e., either no change or decreased) their MVPA. The negative predictive values showed that the SIM correctly classified approximately 35% who do not increase their PA according to accelerometer data.

Inter-measurement reliability for change in physical activity classification between the SIM and accelerometer MVPA-total was relatively poor (k = 0.13, 95%CI: -0.04 to 0.30), and slight to moderate between the SIM and accelerometer MVPA-bouts (k = 0.28, 95%CI: 0.11 to 0.45).

## Discussion

This is the first study to assess the responsiveness of the widely used SIM to detect change in MVPA against accelerometer data when MVPA change was actively promoted. The comparability of responsiveness of the SIM and accelerometer data *(all ≥0*.*57)* demonstrate that the SIM is an acceptable tool to evaluate change in the number of days that people perform ≥30 minutes of MVPA in response to an intervention designed to increase PA. The results provide moderate support for the validity of the SIM to assess physical activity at a single time point using accelerometry as the reference measurement. Additionally, data provide evidence regarding differences between alignment of the SIM and accelerometer data that are analysed in total accumulated MVPA and when MVPA occurred in bouts of ≥10 minutes. Although accuracy of the SIM in classifying increases in change in PA was generally comparable regardless of how accelerometer data were analysed as the reference measurement, there was greater similarity between the SIM and accelerometer data analysed in bouts of ≥10 minutes with responsiveness to change and in the validity at a single time point.

### Agreement between SIM and accelerometry at a single time point

Consistent with previous research [2, 20], the SIM was moderately correlated with the number of days of ≥30 minutes MVPA recorded by accelerometry. Data for the single time point suggest that the SIM is more strongly associated with accelerometer data analysed in 10-minute bouts, where larger correlations existed between the SIM and number of days of ≥30 min MVPA when accelerometer data were analysed in bouts lasting ≥10 minutes (r = 0.51) compared to total minutes of MVPA (r = 0.20). This result aligns with data reported by Milton et al. [20], but contrasts findings presented by Wanner et al. [2]. One potential explanation for lower correlations between the SIM and accelerometer data analysed in total minutes per day is that most of the participants in the present study were administrative staff at a University. Given their occupation, it is likely that these participants accumulated considerable activity in short bouts (less than 10 minutes) during the performance of their typical work-related tasks. These brief bursts of MVPA are unlikely to be detected by the SIM because the SIM asks about PA related to transport and leisure and specifically excludes work-related activities. The exclusion of short duration movements completed at work in the SIM could also explain the finding, observed in this study and by Milton et al. [20], that there was greater similarity in median days of activity between the SIM and MVPA when accelerometer data were analysed in ≥10 minutes bouts than there was between MVPA-total and MVPA-bouts (Table 1).

### Ability of the SIM to detect change in physical activity

The intervention used in the current study resulted in 65% (MVPA-total) and 71% (MVPA-bouts) of participants increasing by one or more days ≥30 minutes MVPA from week one to week two. Based on effect size calculations (SRM and Cohen’s d), responsiveness was moderate to high for the SIM and MVPA-total and MVPA-bouts (all ≥ 0.57). The SRM and Cohen’s d values for the SIM were more closely aligned with values for MVPA-bouts than MVPA-total. These results extend previous findings of comparability between the responsiveness values of longer self-report measures, such as the Active Australia Survey (AAS) and the United States National Health Interview Survey (USNHIS) and device-based measures [25].

Greater similarity between responsiveness values for the SIM and MVPA-bouts than MVPA-total might be explained by methods for promoting PA change in the current intervention and how this aligns with the SIM. Participants were instructed to increase their walking and running over the intervention period and prizes were offered to individuals and teams with the highest increases in accelerometry-derived step counts between assessments. Notwithstanding that some people might have increased incidental walking in bouts of less than 10 minutes, participants were offered access to regular supervised group walking sessions that were supervised by a personal trainer over the period of the intervention. Participation in activities such as the group walking sessions occurred in bouts of 10 minutes or more and this aligns with the SIM measure that includes reference to PA that would typically take place in bouts of 10 or more minutes such as sport, exercise and transport.

With respect to the validity of the SIM to detect change using accelerometer data as the reference measurement small to moderate correlations existed between change in number of days ≥30 minutes MVPA detected by the SIM and accelerometer data for MVPA-total (r = 0.36) and MVPA-bouts (r = 0.40). Although comparisons with other studies should be made with caution given the different measurement properties of self-report scales, our findings compare favourably to the REGICOR (r = 0.26 to 0.34) [27] and Active Australia Survey (r = 0.36) [29] and are slightly lower than that recorded for the GPAQ (r = 0.52) [23], which was based on just 21 participants. Additionally, our results equate favourably with investigations that have compared the IPAQ-SF against accelerometer data in studies that have promoted physical activity change, where there was either an absence of significant correlations between measures [24, 28], or significant correlations were confined to a subcomponent of the IPAQ [26].

Inter-measurement reliability between SIM and accelerometer for change in MVPA from week 1 to week 2 was low when analysed in total minutes per day (k = 0.13) and a fair between SIM and accelerometer when analysed in 10-minute bouts (k = 0.28). Given this is the first study to examine the validity of change in PA using the SIM it is not clear if this finding can be translated to other populations.

The overall accuracy of the SIM to detect increases in PA, where the unit of change is days ≥30 minutes MVPA, was 60% and 63% when compared against MVPA-total and MVPA-bouts of ≥10 min. Therefore, the ability of the SIM to correctly identify people who increased their activity (sensitivity) was moderate. The percentage of people who self-reported increasing PA with the SIM who actually did according to accelerometry (positive predictive value) was high regardless of how accelerometer data were analysed (77% and 78%). The SIM displayed moderate accuracy (48% and 52%), regardless of how accelerometery data were analysed, in correctly classifying people who did not increase their PA.

This study has several limitations that should be considered when interpreting the findings. The sample was not representative of the broader population (i.e., 78% female and recruited through a University staff population). Another potential limitation relates to the use of accelerometer data as the reference measurements for assessing the accuracy of self-report measures. As noted by Troiano et al. [35], Molina et al. [27], and Pedisic & Bauman [6], there is a debate about the use of accelerometry for such purposes based on concerns about generalisability, choice of MVPA cut points, and underreporting of PA in some settings such as cycling and swimming. Nevertheless, in line with Dowd et als. [3] recommendation that choice of PA measures ought to be guided by the intended context for PA measurement, the present investigation was focused specifically on increasing step counts. Further, accelerometer data have been a standard practice in validating self-report measures, particularly where it is not practical to utilise other device-based measures [27] and are typically rated as preferable to self-report measures for most purposes [3, 36, 37].

## Conclusion

The SIM had moderate agreement with accelerometry when days with ≥30 min of MVPA were assessed at single time point. Agreement between the SIM and accelerometry was slight to moderate when assessing improvements in the number of days with ≥30 min of MVPA, as a function of being exposed to an intervention designed to increase PA. Additionally, our data suggested comparability in the responsiveness of the SIM and accelerometer to detect these changes, with most similarity between the SIM and accelerometer data accumulated in bouts. Collectively these data suggest that the SIM has potential as a tool for assessing change in PA settings, such as evaluating the impact of community-based programs designed to increase leisure-based PA, where it might not be possible or feasible to use device-based measures or longer self-report measures.

## Supporting information

S1 Data (XLSX)
